# Caste-biased patterns of brain investment in the subterranean termite *Reticulitermes flavipes*

**DOI:** 10.1016/j.isci.2024.110052

**Published:** 2024-05-22

**Authors:** Austin Merchant, Xuguo Zhou

**Affiliations:** 1 Department of Entomology, Martin-Gatton College of Agriculture, Food and Environment, University of Kentucky, Lexington, KY 40546, USA; 2 Department of Entomology, School of Integrative Biology, College of Liberal Arts & Sciences, University of Illinois Urbana-Champaign, Urbana, IL 61801, USA

**Keywords:** Wildlife behavior, Zoology, Entomology

## Abstract

Investment into neural tissue is expected to reflect the specific sensory and behavioral capabilities of a particular organism. Termites are eusocial insects that exhibit a caste system in which individuals can develop into one of several morphologically and behaviorally distinct castes. However, it is unclear to what extent these differences between castes are reflected in the anatomy of the brain. To address this question, we used deformation-based morphometry to conduct pairwise comparisons between the brains of different castes in the eastern subterranean termite, *Reticulitermes flavipes*. Workers exhibited enlargement in the antennal lobes and mushroom bodies, while reproductives showed increased investment into the optic lobes and central body. In addition, caste-specific enlargement was observed in regions that could not be mapped to distinct neuropils, most notably in soldiers. These findings demonstrate a significant influence of caste development on brain anatomy in termites alongside convergence with eusocial hymenopteran systems.

## Highlights

• Substantial differences in brain allometry are found among termite castes
• Workers show expansion in antennal lobes and mushroom bodies
• Reproductives exhibit expansion in optic lobes and alates in central body
• No sexual dimorphism is observed within castes

## Graphical abstract

**Figure:**
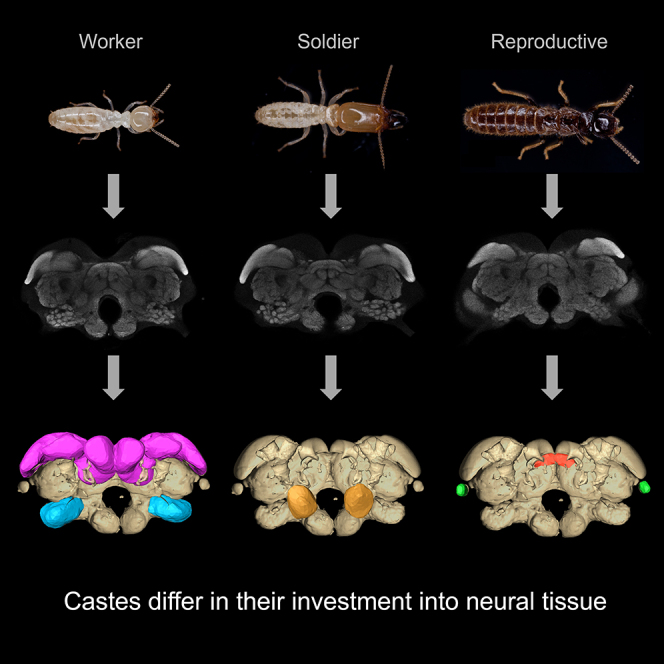

## Introduction

The brain is the center of all behavioral and sensory processes and has remained a popular subject of research for centuries. The size and complexity of an organism’s brain are generally limited by the fact that neural tissue is extremely costly to maintain.^1^^,^^2^^,^^3^ Due in part to these costs, patterns of brain investment differ significantly across the animal kingdom, coinciding with marked differences in sensory and cognitive abilities. A relatively simple example of this phenomenon can be observed in the development of visual versus olfactory sensory systems in day- and night-active insect species. In insects, the primary centers of visual and olfactory processing are the optic lobes and antennal lobes, respectively.^4^^,^^5^ Day-active species often show enlargement in the optic lobes relative to those that are night-active, which often show enlargement in the antennal lobes as an alternative means of navigating low-light environments.^6^^,^^7^^,^^8^^,^^9^ Similar examples of differential investment in these two regions can be observed across *Drosophila* species,^10^ between the paper wasp *Polistes dominula* and its obligate social parasite *Polistes sulcifer*,^11^ and between the ant *Formica fusca* and its obligate social parasite *Polyergus mexicanus.*^12^ These patterns of investment suggest a tradeoff between development of particular regions of the brain, consistent with the idea of neural tissue as energetically costly.

Even within the same species, it is possible to observe significant differences in brain anatomy between individuals. This is especially true in the social insects, which exhibit a division of labor manifested as a caste system. Castes can be broadly categorized as either reproductive or non-reproductive and generally show clear differences in morphology and behavior among one another.^13^ These differences extend to the structure of the brain, although to date the majority of research in this area has focused on the social Hymenoptera. In honeybees and ants, workers show expansion in the sensory-integrating mushroom bodies relative to queens.^14^^,^^15^^,^^16^^,^^17^^,^^18^^,^^19^ This trend is reversed in sweat bees and paper wasps, potentially due to the demands associated with maintaining reproductive dominance in these groups.^20^^,^^21^ Comparisons of workers to soldiers across several ant species have shown expansion of the mushroom bodies in workers, along with expansion in the antennal lobes, which process chemosensory cues.^17^^,^^22^^,^^23^ Significant changes in brain anatomy are also well-characterized within castes, most notably in relation to the nurse-forager transition exhibited by honeybee workers.^24^ Foragers, even those that develop precociously, always possess larger mushroom bodies than nurse bees of any age.^25^ Complex differences in brain allometry have also been described in the leafcutter ant *Atta cephalotes*, which exhibits several worker subcastes: the smallest workers remain in the nest and tend to fungus gardens, intermediate-sized workers harvest leaves outside the nest, and the largest workers fill a defensive role.^26^^,^^27^ Examined together, these and other results indicate that the processes of caste and subcaste development are associated with significant changes in brain structure. Given the role of the brain, these changes are expected to accommodate the specialized task repertoires and sensory demands of different castes and subcastes.^28^

Termites represent another major group of social insects and exhibit complex caste development pathways. In addition to workers and reproductives, termites possess a morphologically distinct soldier caste that is responsible for colony defense. In the lower termites, soldiers differentiate directly from workers rather than following a separate developmental trajectory, as in ants.^29^ The process of worker-to-soldier differentiation requires only two molts but results in a significant change in the appearance and behavior of the individual, with soldiers showing high aggression but an inability to perform any of the tasks that workers are responsible for.^30^ While rare under natural conditions, it is also possible for workers to develop into ergatoids, which function as supplementary reproductives. The sense of vision is of particular note in termites, as workers, soldiers, and ergatoids in all but the most basal termite lineages lack external eyes.^31^ However, alates, which are responsible for dispersing and founding new colonies in which they act as the primary reproductives following insemination, possess functional eyes and wings to aid in their dispersal. Eye and wing development is progressively observed beginning from the nymph stage, which follows a separate trajectory from worker development.^29^

Such extreme differences in sensory abilities and behavior are likely to be reflected in the brain, although to date only a handful of studies have examined this aspect of termite biology. In the dampwood termite *Hodotermopsis sjostedti*, soldiers show expansion in the mandibular motor neurons relative to workers, as well as in distinct clusters of octopaminergic and tyraminergic neurons.^32^ In dampwood termites of genus *Zootermopsis*, reproductives show expansion in the optic lobes relative to non-reproductive castes, while workers show expansion in the antennal lobes and mushroom bodies relative to soldiers and reproductives.^19^^,^^33^ Among non-dampwood termite species, *Reticulitermes speratus* reproductives show expansion in the optic lobes relative to non-reproductive castes,^34^ while *Procornitermes araujoi* workers show expansion in the mushroom bodies relative to soldiers.^35^ These studies are informative, but there is still much that remains a mystery regarding the influence of termite caste development on the brain, particularly as patterns of investment may differ significantly among species with different lifestyles.

The goal of this study was to characterize differences in brain anatomy across castes in the eastern subterranean termite, *Reticulitermes flavipes*. *Reticulitermes flavipes* is a widespread, subterranean termite species that nests within the soil and exploits multiple wood sources at a time using an interconnecting system of tunnels.^36^ This lifestyle contrasts heavily with that displayed by previously studied *Hodotermopsis* and *Zootermopsis* species, which nest within a single piece of wood and notably do not forage. This difference in lifestyle should impose different cognitive demands on individuals, thus influencing the evolution of brain allometry both between and within species. A study of differences in neural tissue investment among castes in termite species exhibiting a variety of lifestyles can therefore lead to a greater understanding of the links between brain allometry and the traits that it influences. With this in mind, we hypothesized that, given the vast differences in behavioral repertoires and sensory requirements between *R. flavipes* castes, we would find differences in neural tissue investment between these castes. To test this hypothesis, we dissected and imaged brains from five different caste phenotypes (workers, soldiers, and three reproductive caste phenotypes: ergatoids, nymphs, and alates), then compared brain anatomy among caste phenotypes using deformation-based morphometry before validating our results manually by measuring and comparing the volumes of distinct brain neuropils.

## Results

### Deformation-based morphometry

**Figure 1:**
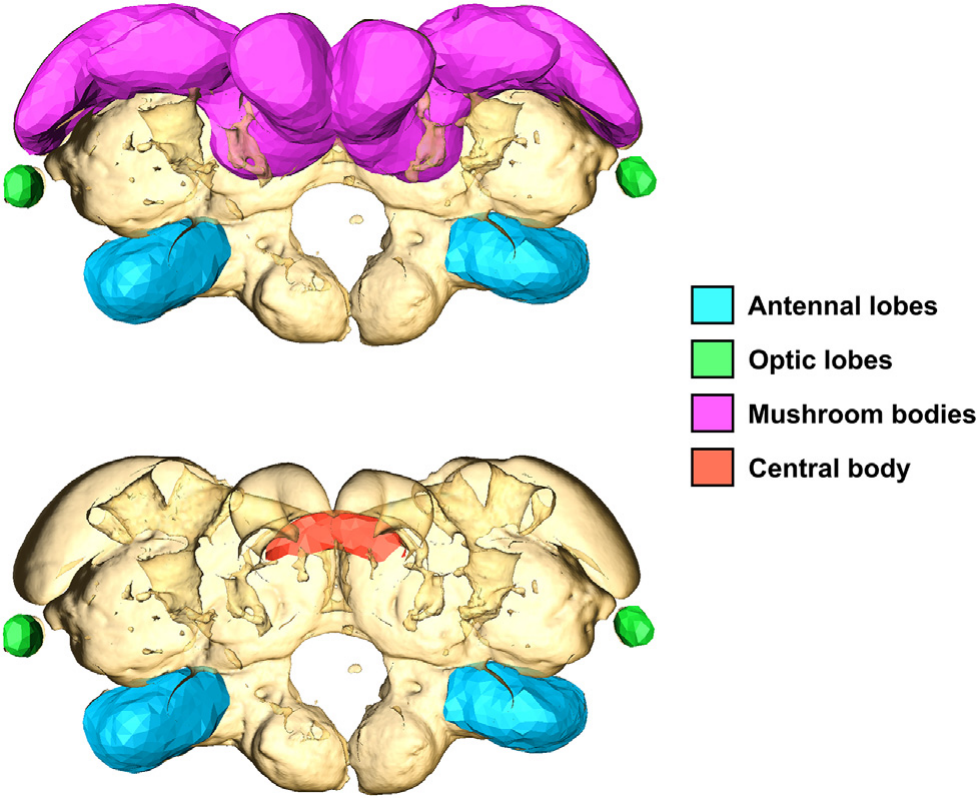
Brain regions measured in validation study Mushroom bodies are not visualized in lower panel so that central body is visible.

**Figure 2:**
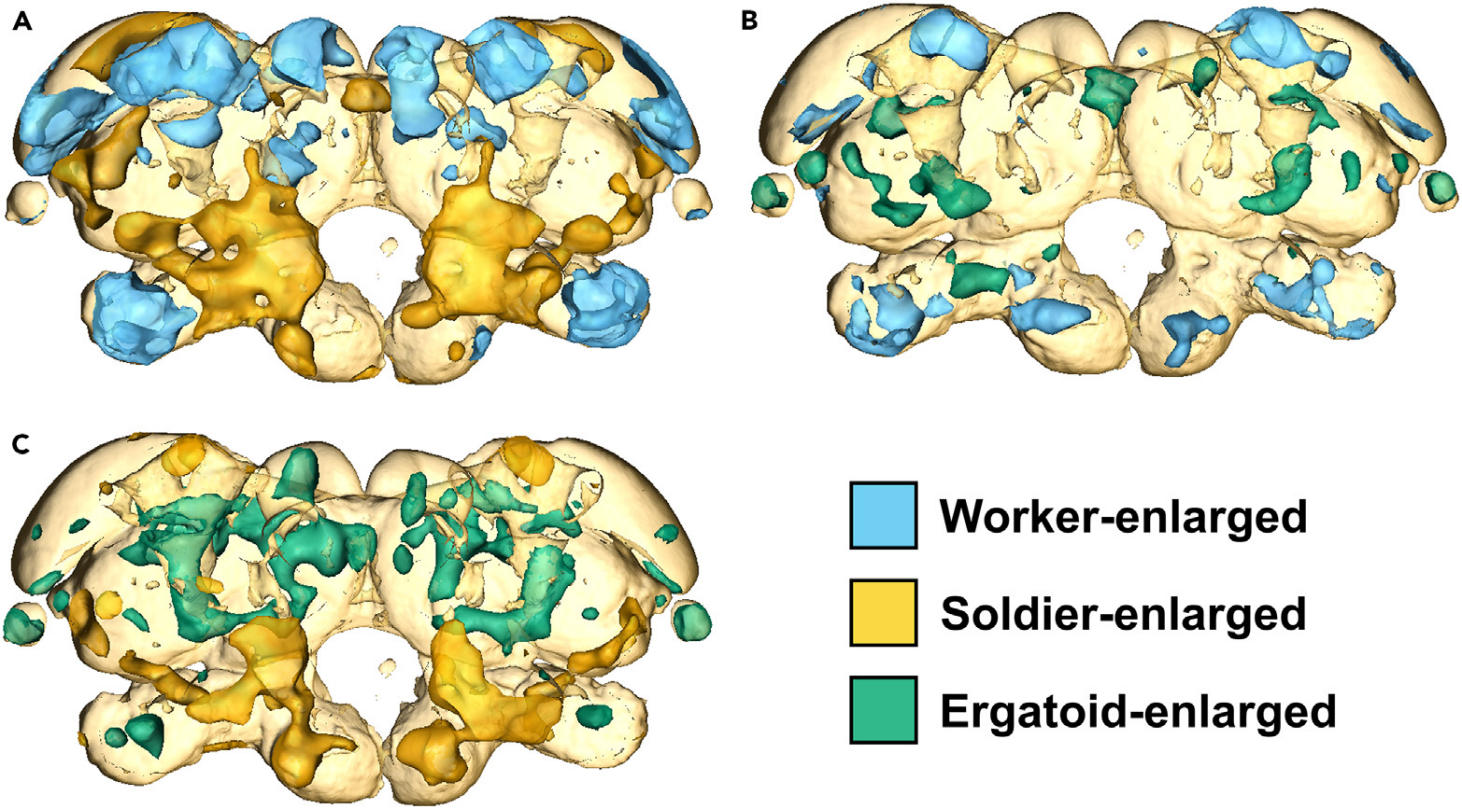
Pairwise DBM-based analysis of worker-derived *R flavipes* castes Colored regions indicate brain regions identified as significantly enlarged in one caste relative to the other (A) Worker-soldier comparison. (B) Worker-ergatoid comparison. (C) Soldier-ergatoid comparison.

Significant differences in brain anatomy were observed between different pairs of termite castes, both generally throughout the brain and in four distinct neuropils: the antennal lobes (ALs), optic lobes (OLs), mushroom bodies (MBs), and central body (CB) (Figure 1; Table 1). First, a series of pairwise comparisons between the three worker-derived castes (workers, soldiers, and reproductives, represented as ergatoids) was carried out (Figure 2). The ALs and MBs showed enlargement in workers relative to both soldiers and ergatoids. The OLs showed enlargement in ergatoids relative to both workers and soldiers. Finally, enlargement at or close to the central complex (CX) was identified in soldiers and ergatoids relative to workers. Although other regions showing caste-specific enlargement were identified, these regions could not be mapped to distinct neuropils.

**Table 1:** Summary of number of brains and template used for each pairwise comparison

Template brain	Group 1	N	Group 2	N
Worker	Worker	60	Soldier	59
Worker	Worker	30	Ergatoid	30
Worker	Soldier	30	Ergatoid	30
Worker	Worker	30	Nymph	30
Worker-Alate	Worker	40	Alate	44
Alate	Nymph	30	Alate	30
Worker	Male Worker	41	Female Worker	33
Worker	Male Soldier	26	Female Soldier	33
Worker	Male Ergatoid	15	Female Ergatoid	15
Nymph	Male Nymph	16	Female Nymph	14
Alate	Male Alate	21	Female Alate	23

**Figure 3:**
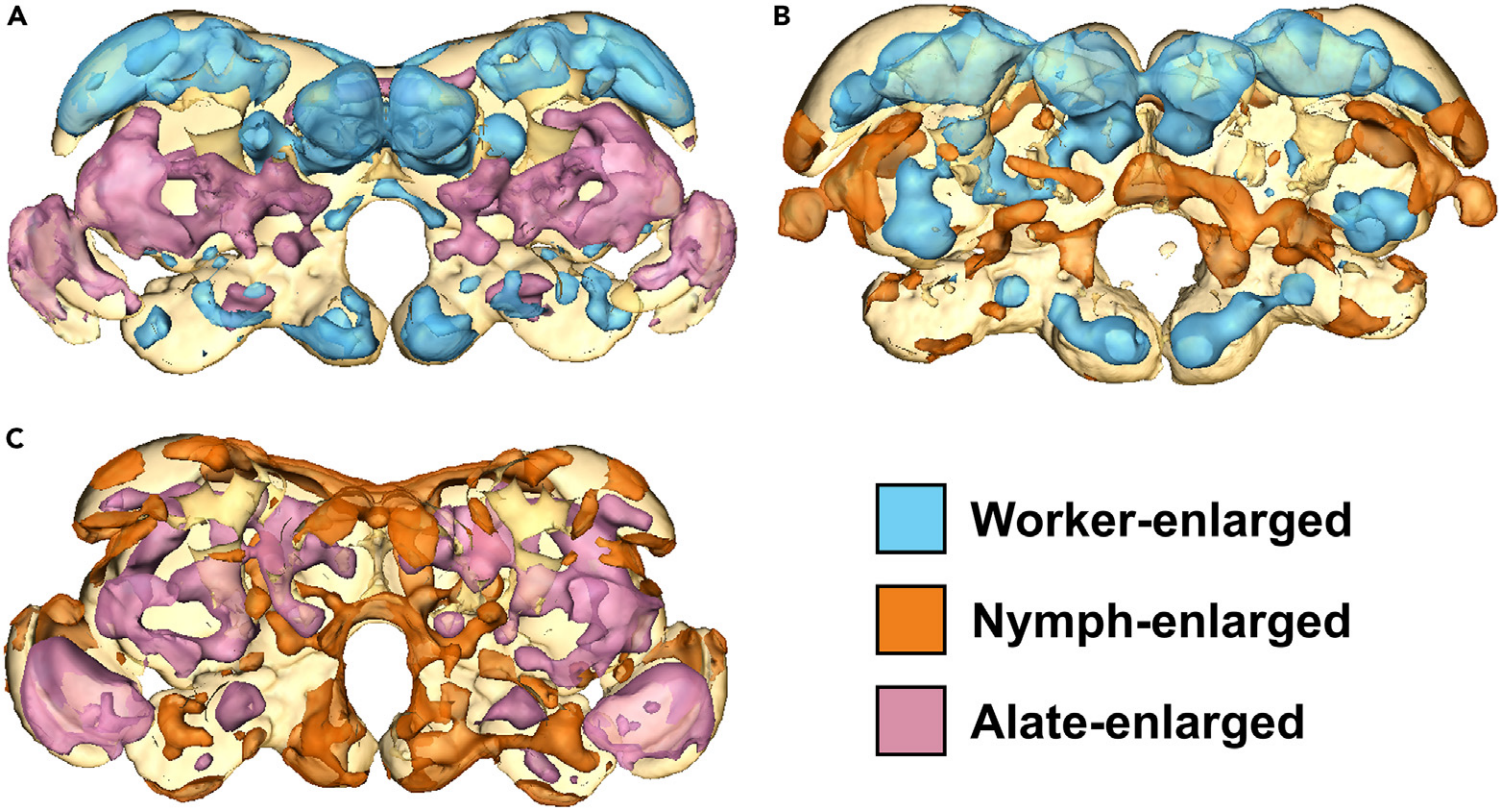
Pairwise DBM-based analysis of *R flavipes* workers, nymphs, and alates Colored regions indicate brain regions identified as significantly enlarged in one caste phenotype relative to the other (A) Worker-nymph comparison. (B) Worker-alate comparison. (C) Nymph-alate comparison.

Next, a series of pairwise comparisons between workers, nymphs, and alates was carried out (Figure 3). The ALs showed enlargement in both workers and nymphs relative to alates, while the MBs showed enlargement in workers relative to both nymphs and alates. The OLs showed enlargement in nymphs and alates relative to workers and in alates relative to nymphs. Enlargement at or close to the CX was identified in nymphs and alates relative to workers. As in the comparisons of the worker-derived castes, other regions showing phenotype-specific enlargement were identified but could not be mapped to distinct neuropils.

**Figure 4:**
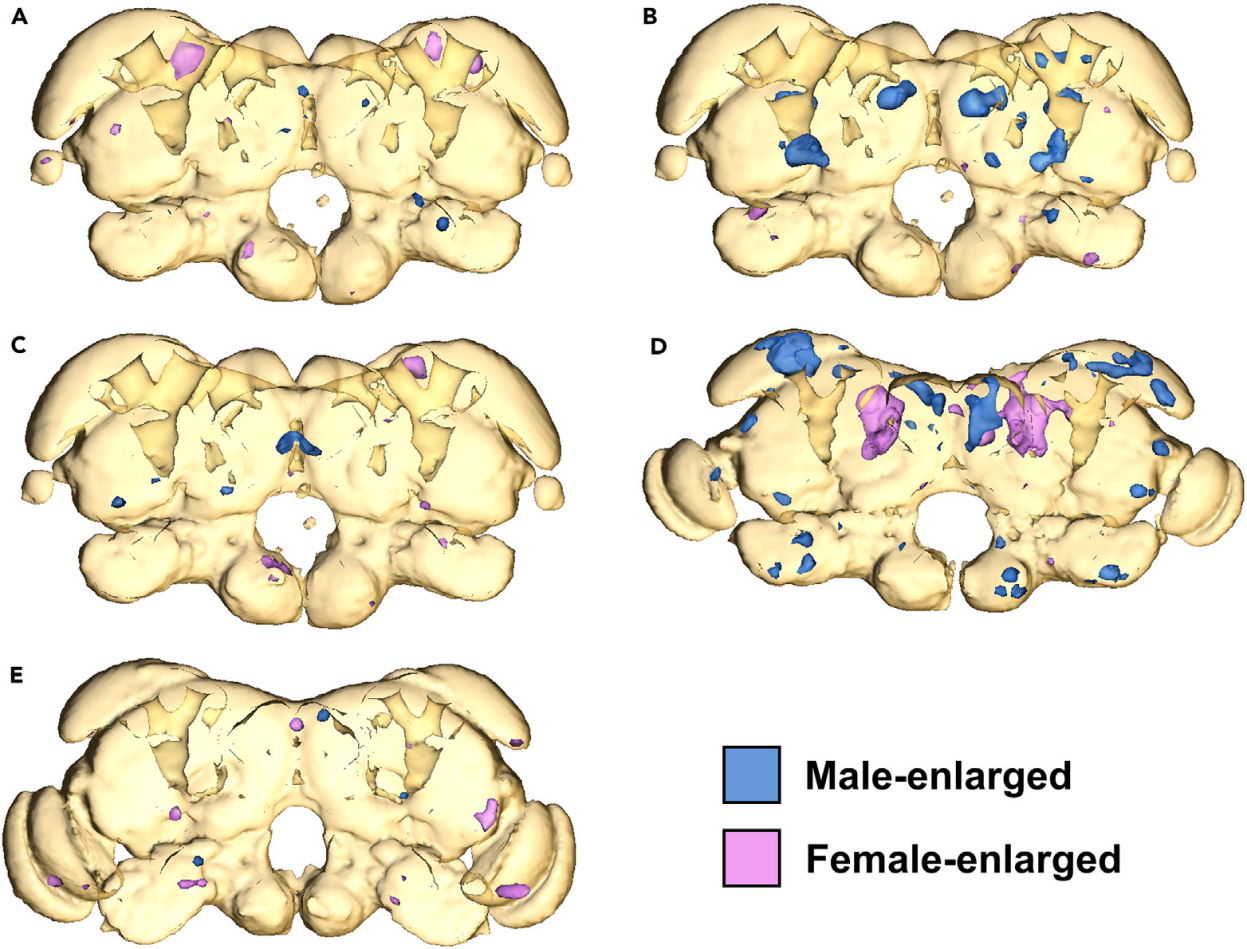
Pairwise DBM-based analysis of sexual dimorphism among *R flavipes* caste phenotypes Colored regions indicate brain regions identified as significantly enlarged in one sex relative to the other (A) Worker comparison. (B) Soldier comparison. (C) Ergatoid comparison. (D) Nymph comparison. (E) Alate comparison.

Finally, the presence of sexual dimorphism within castes was tested in all five caste phenotypes by comparing male and female brains (Figure 4). Sex had no effect on brain anatomy within caste phenotypes.

### Validation

**Figure 5:**
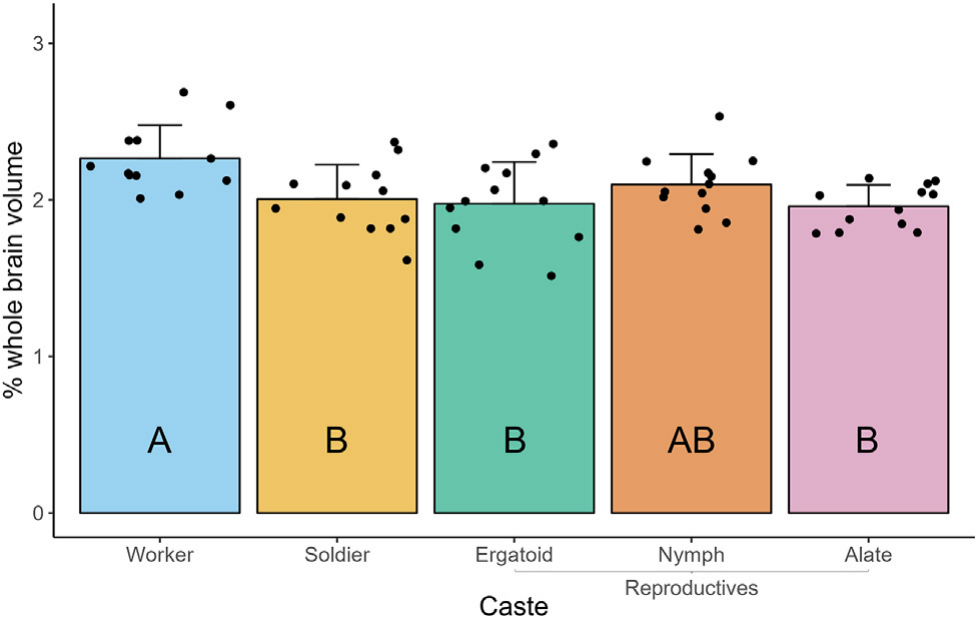
Comparison of antennal lobe (AL) volume among *R flavipes* caste phenotypes Volume of each AL is expressed as a percent of whole brain volume to account for individual variation in brain size. AL volume was measured in 6 individuals per phenotype; left and right ALs were measured separately to generate 12 measurements per phenotype. Individuals of all phenotypes except alates were pooled from a total of five colonies and randomly selected for validation, while all alates were collected from one colony. Bars represent mean +SD, while points represent individual ALs. Letters denote significance groups as determined using a one-way ANOVA followed by Tukey’s post-hoc test, where *p* < 0.05 was used as the threshold for significance.

Following the deformation-based morphometry (DBM) analysis, validation was carried out by manually measuring the volumes of distinct, identifiable neuropils in the brains of termites from each caste phenotype. A significant effect of caste phenotype on AL volume was observed (Figure 5; one-way ANOVA; F(4,55) = 4.336, *p* < 0.05). The ALs of workers each comprised 2.265 ± 0.212% (average ±SD) of the brain’s total volume, which was significantly greater than that of soldiers (2.006 ± 0.220%), ergatoids (1.976 ± 0.267%), and alates (1.959 ± 0.136%) but not nymphs (2.098 ± 0.195%).

**Figure 6:**
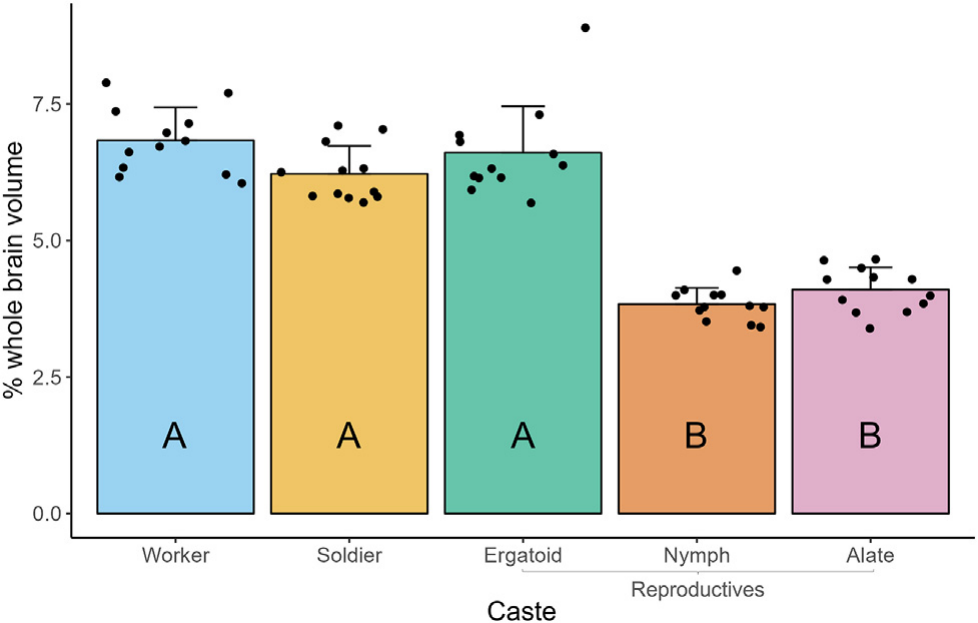
Comparison of mushroom body (MB) volume among *R flavipes* caste phenotypes Volume of each MB is expressed as a percent of whole brain volume to account for individual variation in brain size. MB volume was measured in 6 individuals per phenotype; left and right MBs were measured separately to generate 12 measurements per phenotype. Individuals of all phenotypes except alates were pooled from a total of five colonies and randomly selected for validation, while all alates were collected from one colony. Bars represent mean +SD, while points represent individual MBs. Letters denote significance groups as determined using a one-way ANOVA followed by Tukey’s post-hoc test, where *p* < 0.05 was used as the threshold for significance.

A significant effect of caste phenotype on MB volume was observed (Figure 6; one-way ANOVA; F(4,55) = 77.32, *p* < 0.05). The MBs of workers (6.833 ± 0.606%), soldiers (6.220 ± 0.510%), and ergatoids (6.610 ± 0.849%) were significantly larger than those of nymphs (3.833 ± 0.297%) and alates (4.099 ± 0.409%).

**Figure 7:**
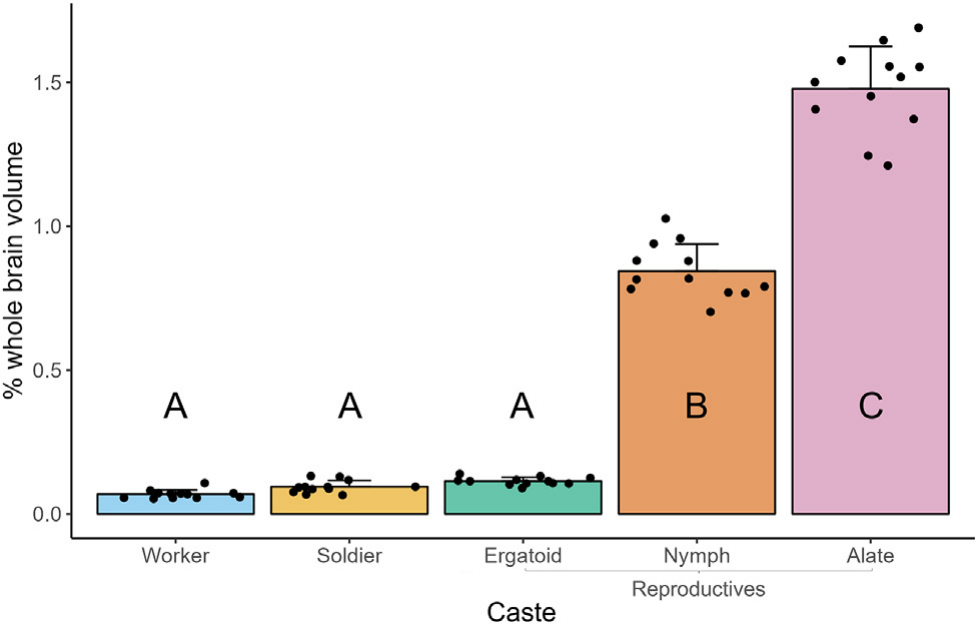
Comparison of optic lobe (OL) volume among *R flavipes* caste phenotypes Volume of each OL is expressed as a percent of whole brain volume to account for individual variation in brain size. OL volume was measured in 6 individuals per phenotype; left and right OLs were measured separately to generate 12 measurements per phenotype. Individuals of all phenotypes except alates were pooled from a total of five colonies and randomly selected for validation, while all alates were collected from one colony. Bars represent mean +SD, while points represent individual OLs. Letters denote significance groups as determined using a one-way ANOVA followed by Tukey’s post-hoc test, where *p* < 0.05 was used as the threshold for significance.

A significant effect of caste phenotype on OL volume was observed (Figure 7; one-way ANOVA; F(4,55) = 748.3, *p* < 0.05). The OLs of nymphs (0.844 ± 0.094%) and alates (1.477 ± 0.147%) were significantly larger than those of workers (0.068 ± 0.015%), soldiers (0.095 ± 0.022%), and ergatoids (0.114 ± 0.013%).

**Figure 8:**
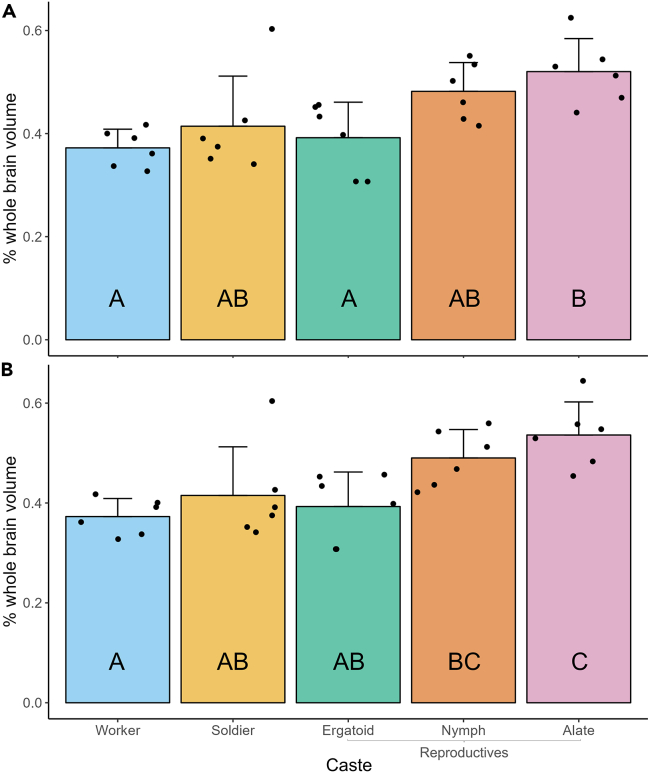
Comparison of central body (CB) volume among *R flavipes* caste phenotypes Volume of each CB is expressed as a percent of whole brain volume to account for individual variation in brain size. CB volume was measured in 6 individuals per phenotype. Individuals of all phenotypes except alates were pooled from a total of five colonies and randomly selected for validation, while all alates were collected from one colony. Bars represent mean +SD, while points represent individual CBs. Letters denote significance groups as determined using a one-way ANOVA followed by Tukey’s post-hoc test, where *p* < 0.05 was used as the threshold for significance. (A) CB volume expressed as a percent of whole brain volume without adjustment. (B) CB volume expressed as a percent of whole brain volume minus the volume of the optic lobes.

Finally, a significant effect of caste phenotype on CB volume was observed (Figure 8A; one-way ANOVA; F(4,55) = 5.166, *p* < 0.05). The CBs of alates (0.5203 ± 0.064%) were significantly larger than those of workers (0.372 ± 0.036%) and ergatoids (0.392 ± 0.069%) but not soldiers (0.414 ± 0.097%) or nymphs (0.482 ± 0.056%). When OL-adjusted data were used, these relationships between CB volume showed slight change—the CBs of alates (0.536 ± 0.066%) were significantly larger than those of workers (0.373 ± 0.036%), soldiers (0.415 ± 0.097%), and ergatoids (0.393 ± 0.069%), while the CBs of nymphs (0.490 ± 0.057%) were significantly larger than those of workers (Figure 8B).

## Discussion

### Workers showed expansion in the antennal lobes and mushroom bodies

Our analysis revealed diverse patterns of caste-specific brain enlargement between *R. flavipes* termite caste phenotypes. Workers comprise the majority of a termite colony and consistently showed enlargement in two key brain regions, the ALs and the MBs. The ALs are the primary olfactory centers of the insect brain and receive input directly from olfactory sensory neurons located along the antennae,^5^ while the MBs act as centers for multisensory integration, learning, and memory.^37^ Both of these regions commonly appear as showing caste-specific enlargement in eusocial insects, most often in the worker caste relative to reproductives and/or defensive castes or subcastes. Species in which this is the case include the honeybee *Apis mellifera*,^14^^,^^15^^,^^16^ the turtle ant *Cephalotes varians*,^17^ the carpenter ant *Camponotus ocreatus*,^18^ the leafcutter ant *Atta cephalotes*,^26^ those of the army ant genus *Eciton*,^22^ those of the ant genus *Pheidole*,^23^ and those of the termite genera *Zootermopsis* and *Procornitermes*.^19^^,^^35^ In species that show this pattern of investment, workers possess complex behavioral repertoires that may require stronger cognitive processing capabilities relative to other castes. When the inverse is true, as in the sweat bee *Augochlorella aurata*,^20^ reproductives may face unique cognitive challenges not encountered by the worker caste.

In the case of *R. flavipes*, workers are responsible for an array of tasks, which includes foraging, nest construction and repair, and brood care. By contrast, the repertoires of the remaining castes are highly restricted. Soldiers are responsible for defending the colony from attack, while alates and ergatoids are responsible for reproduction.^29^ Considering the reduced number and complexity of tasks that non-worker castes are responsible for, a low demand for investment into neural tissue might be expected. The role of nymphs in the colony is less clear. In *Coptotermes formosanus,* nymphs are capable of feeding themselves and caring for soldiers when workers are absent,^38^ although it is unknown if nymphs contribute labor under field conditions. Nymphs are also occasionally found among foraging populations, although it has been suggested that they are feeding themselves in this case rather than aiding in foraging.^39^ Nevertheless, nymphs at least appear to be able to perform some tasks associated with the worker repertoire. That AL volume was not found to significantly differ between workers and nymphs may reflect an increased dependency on chemical communication in these two phenotypes relative to soldiers and sexually mature reproductives.

Correlations between task complexity and MB volume have been documented within the eusocial Hymenoptera, particularly in the case of the age polyethism exhibited by honeybee workers. Young workers remain within the hive and act as nurses, while older workers regularly exit the hive and act as foragers.^40^ In the case of honeybees, foraging is a complex task that requires strong navigational ability and the ability to recognize and remember the locations of nectar resources. Foragers show expansion in MB volume relative to nurses at all ages, even when foraging is induced precociously.^25^^,^^41^ A similar trend is observed in the leafcutter ant *Atta cephalotes,* in which medium-sized workers that exhibit a wide behavioral repertoire possess larger MBs than those specialized toward certain tasks, such as fungus garden tending or nest defense.^26^ Likewise, expansion of the ALs, as is observed in *Eciton* army ant and *Zootermopsis* termite workers,^19^^,^^22^ suggests increased sensitivity to chemosensory cues, including the pheromones that constitute a major portion of termite communication. Interestingly, only the MBs were found to be enlarged in workers relative to soldiers in the higher termite *Procornitermes araujoi*,^35^ although the reason why is unclear. Overall, expansion of these regions in eusocial insects appears to correlate with an increase in task complexity or task repertoire size, or both.

Based on the results of our validation, the ALs of workers were approximately 8% and 16% larger than those of nymphs and alates, respectively, when whole brain volume was taken into account (Figure 5), while their MBs were approximately 78% and 67% larger (Figure 6). Worker ALs were approximately 13% and 15% larger than those of soldiers and ergatoids, respectively (Figure 5), while their MBs were approximately 10% and 3% larger (Figure 6). Given that soldiers and ergatoids differentiate directly from workers in *R. flavipes*, the possible magnitude of change in a particular brain region may be physiologically constrained, despite any theoretical differences in cognitive function between castes. Worker and nymph/alate development separate into different trajectories at the third instar,^29^ providing a much earlier origin point and greater length of time for specializations in regional brain volume to develop.

### Reproductives showed expansion in the optic lobes and central body

We examined three phenotypes of the reproductive caste, two of which—nymphs and alates—follow a separate developmental trajectory from workers, while one—ergatoids—differentiates directly from workers. Nymphs and alates showed significant expansion in the OLs, which are involved in visual processing,^4^ relative to all worker-derived castes. The largest difference was observed between alates and workers, in which an approximately 22-fold difference in OL volume was recorded (Figure 7). Our DBM analysis identified expansion in ergatoid OLs relative to those of workers and soldiers, although the results of our validation did not confirm this significance. However, the OLs of ergatoids were approximately 67% and 20% larger than those of workers and soldiers, respectively (Figure 7).

**Figure 9:**
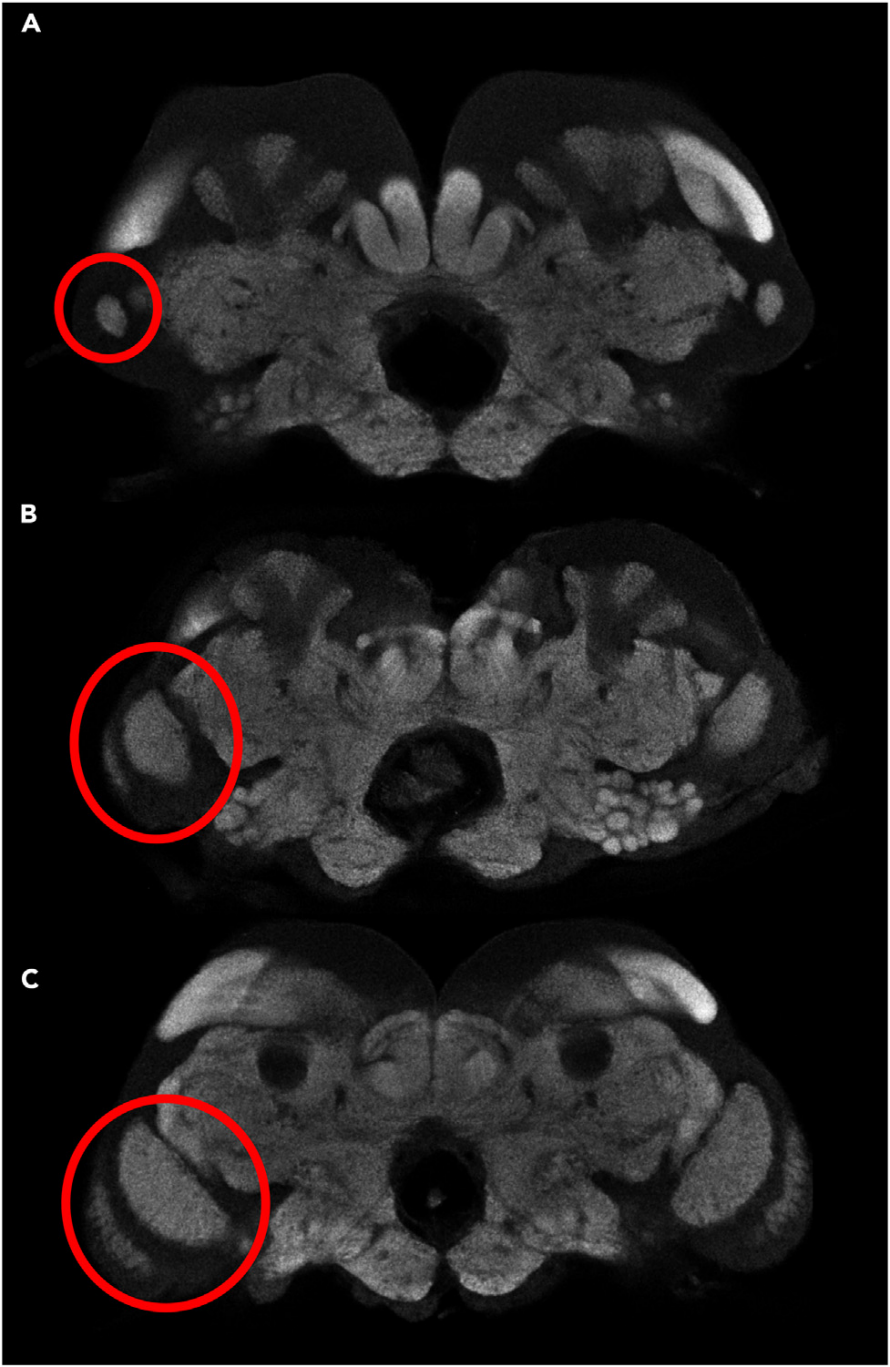
Visual comparison of optic lobes Caste phenotypes used in this study included worker (A), nymph (B), and alate (C). In each case, the optic lobe was highlighted by red circle.

Alates are the only termite caste phenotype that possesses functional eyes and wings. In *R. flavipes*, mature colonies produce alates throughout winter, which disperse in spring to search for mates and find new colonies.^42^ Shortly after pairing, the alates lose their wings and retreat underground into total darkness. Despite the transient usage of eyes in termites, they are integral to the process of colony foundation. Therefore, it is expected that alates would invest significantly into optic sensory systems, while the remaining castes would not. The OLs of the worker-derived castes show close to zero development relative to those of nymphs and alates (Figure 9). Interestingly, the OLs of ergatoids were, on average, larger than those of workers and soldiers. It is possible that the process of sexual maturation in *R. flavipes* is, to an extent, coupled with development of visual sensory systems. Although *R. flavipes* ergatoids do not exhibit any external eye development, ergatoids with vestigial eyes are observed in termites from the genera *Nasutitermes* and *Mastotermes*^43^^,^^44^^,^^45^^,^^46^ and in the family Termitidae.^47^ Maekawa et al. noted a lack of external eye development in ergatoids of the congeneric *Reticulitermes speratus*.^48^ It is possible that in ergatoids of this species, as well, only minor enlargement of the OLs occurs without compound eye development. Our DBM analysis also identified regions of caste-specific enlargement close to the OLs in nymphs, alates, and ergatoids. These regions may include portions of the lateral protocerebrum to which OL neurons project, as is observed in other insects,^49^^,^^50^ although further investigation is necessary to validate and determine the source of this enlargement.

We also observed expansion in the CBs of nymphs and alates relative to those of the worker-derived castes, particularly workers. The CBs of nymphs and alates were approximately 31% and 44% larger than those of workers, respectively (Figure 8). The CB, along with the protocerebral bridge and paired noduli, comprises the CX.^51^ The CX, as a whole, is involved in a number of behavioral processes but notably functions as the center of sensory-motor integration in the insect brain.^52^ It is possible that expansion of the CB in alates and, by extension, nymphs is in some way related to the complex task of flight that only they are capable of. Investment into the CX in general has also been suggested to improve navigation ability in dark, subterranean environments in ants.^26^ Given that many extant termite genera, such as *Zootermopsis*, nest within a single piece of wood and thus exhibit a simple nest structure in comparison to *R. flavipes* and other subterranean termites, it would be of future interest to investigate whether relative CX investment differs in termite species exhibiting different levels of nest complexity.

### Regional expansion in soldiers

Soldiers are significantly more aggressive than workers, and in *R. flavipes* the transition from worker to soldier is accomplished in just two molts.^29^ In addition to the loss of AL and MB volume discussed previously, expansion in other regions of the brain is likely necessary to complete the transition to the soldier behavioral syndrome. Our DBM analysis identified large regions of soldier-specific expansion close to the ALs relative to both workers and ergatoids. Although this region could not be mapped to a distinct neuropil, it overlaps with a population of neurons previously identified as showing soldier-specific expansion in the dampwood termite *Hodotermopsis sjostedti*.^32^ It was suggested by the authors of this study that this neuron population may be involved in modulating the aggressive responses of soldiers, citing research in locusts in which an analogous population of neurons was demonstrated to influence responses to stressful stimuli.^53^ A second possibility is that this region contains neurons that project to the subesophageal zone (SEZ). In termites, the SEZ is separate from the central brain and is enlarged in soldiers relative to workers.^54^ Specifically, the SEZ contains mandibular motor neurons, which control movement of the mouthparts and are likewise enlarged in soldiers. Further study is necessary to confirm the source of soldier-specific expansion, as well as its potential function.

### Sexual dimorphism was not observed in any caste

In addition to comparing brain anatomy between termite castes, we tested for the possibility of sexual dimorphism in brain anatomy within castes. Unlike the eusocial Hymenoptera, which follow a haplodiploid sex determination system and exhibit colonies composed exclusively of females, termites are diploid and each caste is generally made up of both male and female individuals.^55^ Our DBM analysis did not identify sexual dimorphism within any of the five caste phenotypes observed in this study. This result is not unexpected, as neither *R. flavipes* nor termites in general are known to show sexual dimorphism in relation to behavior. There are, however, other forms of sexual dimorphism exhibited among termites that could potentially influence brain anatomy. Sexual size dimorphism is observed in the workers of many termite species, which in turn leads to sex-specific biases in the soldier caste, or to soldier castes consisting of a single sex.^56^ Alates also tend to show sexual size dimorphism, with females being larger than males.^57^^,^^58^ Overall, these differences within castes may be too minor to produce significant changes in brain anatomy, although investigation into a larger variety of termite species would be necessary to confirm this.

### Limitations of the study

Here, we have provided a broad overview of the major differences in brain anatomy observed between *R. flavipes* castes. Structural changes observed in workers relative to the other castes show convergence with the eusocial Hymenoptera, while reproductives show clear development of the visual sensory system. The functions of other identified regions of enlargement, such as those observed in soldiers, are more speculative. However, these results provide a foundation for future work intending to establish a causal link between caste-specific enlargement of the brain and caste-specific behaviors. Comparative study between termite species is also of interest as more information in this area becomes available, as termites exhibit a wide array of lifestyles that may be associated with different cognitive demands. Relative to *R. flavipes*, single-piece nesting genera such as *Zootermopsis* do not need to forage because they live within their food source. Likewise, open-air foraging is observed within some termite species,^59^^,^^60^^,^^61^ which may introduce additional navigational complexity to the task of foraging.

Notably, we were not able to include primary reproductives in our study due to a lack of availability. After colony foundation, the founding alates—now the colony’s primary reproductives—undergo a number of behavioral and physiological changes to suit their new role, which may be accompanied with changes in the brain. In addition to enlargement of the gonads,^62^ primary reproductives exhibit a negative phototaxis and gradual degeneration of the compound eyes,^63^^,^^64^ which has shown to be associated with OL degradation and loss of visual acuity in the congeneric *Reticulitermes speratus*.^34^

Overall, the differences that we have observed contribute to a larger picture of how behavior may influence neural tissue investment. Termites represent a case in which the tasks that an individual organism is generally responsible for, including foraging, reproduction, and self-defense, are decoupled, and distributed among behaviorally specialized castes. How these different patterns of investment may have evolved and whether they remain consistent across the termite clade represent questions that can be elucidated through future work.

## STAR★Methods

### Key resources table

**Table:** 

REAGENT or RESOURCE	SOURCE	IDENTIFIER
**Antibodies**		

Mouse monoclonal anti-Bruchpilot (nc82)	Developmental Studies Hybridoma Bank	Cat#nc82; RRID: AB_2392664
Goat anti-mouse IgG, Alexa Fluor 546	Thermo Fisher Scientific	Cat#A-11030; RRID: AB_2534089

**Chemicals, peptides, and recombinant proteins**		

Triton X-100	Thermo Fisher Scientific	Cat#85111
Paraformaldehyde (PFA)	Thermo Fisher Scientific	Cat#28908
Normal goat serum	Thermo Fisher Scientific	Cat#50197Z
Poly-L-lysine hydrobromide	Sigma-Aldrich	Cat#P1524-25MG
Kodak Photo-Flo 200 solution	Kodak	Cat#1464510
Xylene	Thermo Fisher Scientific	Cat#9990501
Ethyl alcohol, 200 proof	Sigma Aldrich	Cat#EX0276-4
DPX mountant	Thermo Fisher Scientific	Cat#X1525

**Deposited data**		

Confocal data used in pairwise comparisons	This paper	https://doi.org/10.5061/dryad.m37pvmd9x

**Software and algorithms**		

Amira v2020.1	Thermo Fisher Scientific	RRID:SCR_007353
R v4.1.1	R Core Team	RRID:SCR_001905
Computational Morphometry Toolkit (CMTK)	https://www.nitrc.org/projects/cmtk/	RRID:SCR_002234
Fiji	https://fiji.sc/	RRID:SCR_002285

### Resource availability

#### Lead contact

Further information and requests for resources and reagents should be directed to and will be fulfilled by the lead contact, Xuguo Zhou (xgzhou@illinois.edu).

#### Materials availability

This study did not generate new unique reagents.

#### Data and code availability

• Confocal data have been deposited at Dryad and are publicly available as of the date of publication. DOI is listed in the key resources table.
• This paper does not report original code.
• Any additional information required to reanalyze the data reported in this paper is available from the lead contact upon request.

### Experimental model and study participant details

#### Reticulitermes flavipes

*Reticulitermes flavipes* termite colonies were collected from two separated sites at Red River Gorge within the Daniel Boone National Forest, KY, USA (R-I: 37° 47′ 38″ *N* 83° 35′ 55″ W and R-II: 37° 47′ 20″ *N* 83° 35′ 42″ W, respectively) in the summer of 2021. Except for alates, termites were collected using moistened cardboard rolls (diameter = 8.5 cm, height = 15.2 cm, wrapped by a rubber band) placed under logs, which served as bait traps. Once transported to the lab, termites were reared in plastic boxes of dimensions 32 × 10 × 22 cm (length x height x width) stocked with moistened pine wood mulch. Boxes were placed in a 27 ± 1°C incubator and maintained in 24-h darkness. Alates were collected by hand and placed inside plastic tubes, then used in dissections the following day. A total of 5 colonies were used in this study. Workers, soldiers, and nymphs were found in all 5 colonies, while only 1 colony possessed alates, which were collected during a swarming event. Given that workers and nymphs may belong to one of several different instars, which may influence brain development, we attempted to control for the influence of age by only using individuals of approximately the same body size.^65^^,^^66^

Ergatoids were induced separately using a model orphaning assay.^67^ Groups of 100 workers of approximately the same size were collected from a single colony and placed inside a 5.5-cm diameter Petri dish containing a moistened paper towel disc. Petri dishes were sealed with Parafilm, then placed in a 27 ± 1°C incubator in total darkness. Beginning from 1 month following setup, Petri dishes were observed every other day for ergatoid differentiation. Ergatoids were identified by morphology and sexed, then returned to the Petri dish until an ergatoid of the opposite sex differentiated. Once an ergatoid of each sex had differentiated, one of the two ergatoids was removed from the Petri dish and placed in a smaller 2.5-cm Petri dish containing 10 nestmate workers. Once approximately 20 ergatoids of each sex had been collected, dissection of all ergatoids was carried out in a single day. Approximately 3 months passed from setup of the orphaning assay to the date of ergatoid dissection.

### Method details

#### Brain dissection, fixation, and imaging

*Reticulitermes flavipes* brains were collected and fixed using a previously described antibody staining protocol,^68^ with adjustments. Termites were anesthetized on ice, then dissected in PBS. Brains were fixed in 2% paraformaldehyde (PFA) for 55 min, then washed in PBS mixed with 10% Triton X-100 (PBT) three times for 15 min each. Next, brains were incubated in PBT containing 5% normal goat serum (NGS; Thermo Fisher Scientific; Waltham, MA, USA) for 1 h, then incubated overnight at 4°C in PBT containing 5% NGS and the primary antibody, mouse anti-nc82 (Developmental Studies Hybridoma Bank; Iowa City, IA, USA), at 1:50. The following day, brains were washed in PBT three times for 15 min each, then incubated overnight in PBT containing 5% NGS and the secondary antibody, Alexa Fluor 546 goat anti-mouse IgG (Thermo Fisher Scientific), at 1:500.

The following day, brains were fixed in 4% PFA for 4 hours, washed in PBT three times for 15 min each, then mounted onto a coverslip coated in a poly-L-lysine solution. The poly-L-lysine solution was generated by mixing 25 mg of poly-L-lysine hydrobromide (Sigma-Aldrich; St. Louis, MO, USA) with 2 mL of Invitrogen Ultrapure distilled water (Thermo Fisher Scientific) until the powder had dissolved, then transferring the mixture to a 50 mL conical vial, adding another 30 mL of distilled water, and then adding 64 μL of Kodak Photo-Flo 200 Solution (Kodak; Rochester, NY, USA). After mounting them onto the coverslip, brains were dehydrated in graded ethanol (30%, 50%, 70%, 95%, 100%, 100%, 100%) for 5 min per step, then cleared in xylene (100%, 100%, 100%) for 5 min per step. Finally, the coverslip was placed on a slide treated with several drops of DPX (Thermo Fisher Scientific) and ventilated in darkness within a fume hood for at least 2 days prior to imaging.

Images of *R. flavipes* brains were acquired using a Leica SP8 DLS laser scanning confocal microscope at the Arts & Sciences Imaging Center at the University of Kentucky. Whole brains were imaged at 1024 × 1024 pixel resolution using a 10× dry objective (HC PL APO 10×/0.40). Image stacks were generated by capturing images of brains at 1 μm intervals, which were then saved as.TIF files.

#### Template brain generation

Template brain generation was carried out using the registration software Computational Morphometry Toolkit (CMTK; https://www.nitrc.org/projects/cmtk/). Using Fiji (https://fiji.sc/), image stacks were rotated so that all were oriented in the same direction, and the number of images per stack was normalized to 150. Processed image stacks were then exported as NRRD files. Shape-averaged template brains were generated using CMTK’s *iterative_shape_averaging* function. For the worker, nymph, and alate templates, individual brains of the corresponding caste were rated on the bases of symmetry and overall shape, and the 5 highest-rated brains of each sex were selected for use in template generation. A worker-alate intercaste template brain was also generated by averaging the worker and alate templates, as the difference in optic lobe size between these two castes was too large to perform a suitable comparison using the worker template.

#### Deformation-based morphometry

Deformation-based morphometry (DBM) was carried out using CMTK. The following pairwise comparisons were performed: worker-soldier, worker-ergatoid, soldier-ergatoid, worker-nymph, worker-alate, and nymph-alate. In addition, pairwise comparisons were performed between males and females of the worker, soldier, ergatoid, nymph, and alate caste phenotypes. A summary of the number of brains and template used for each comparison is presented in Table 1. For each pairwise comparison, nonrigid registration of each individual brain to the corresponding template was performed using CMTK’s *registration*, *affine*, and *reformatx* functions, resulting in a Jacobian file as output. Jacobian files indicated the degree of per-voxel expansion or shrinkage exhibited in an individual brain relative to the template, expressed as a numerical value. Specifically, voxels with values < 1.0 indicated regions of lesser volume in the individual brain than in the template, while those with values > 1.0 indicated regions of greater volume in the individual brain than in the template.

Next, Jacobian files were downsampled using a custom Fiji script provided by S.C. (personal communication). Jacobian files from each caste or sex group were then compared to one another by performing a per-voxel t-test using CMTK’s *ttest* function. Significant threshold t-values were determined in R (v4.1.1; https://www.r-project.org/) by performing permutation tests. Each test was repeated 10,000 times and quantiles of 2.5% and 97.5% were selected as threshold t-values to use in data visualization. Data were visualized in Amira (v2020.1, Thermo Fisher Scientific).

### Quantification and statistical analysis

In order to validate the results of the DBM analysis, the volumes of visually distinct neuropils were measured in individual termite brains. A total of 3 male and 3 female brains from each of the worker, soldier, ergatoid, nymph, and alate caste phenotypes were randomly selected and imported into Amira as.NII files. The antennal lobes (ALs), optic lobes (OLs), mushroom bodies (MBs), and central body (CB) of each individual brain were manually selected using Amira’s Segmentation Editor, then their volumes were calculated (Figure 1). Segmentation was performed by the same individual for all brains. The volume of the whole brain was also measured and used to express the volume of each neuropil as a percent of total brain volume. Due to the extreme difference in OL size between nymphs/alates and workers/soldiers/ergatoids, an additional set of measurements was generated by dividing the volume of each region by the volume of the whole brain after subtracting the volume of the OLs. Measurements generated using this method are referred to as “OL-adjusted” and are only used where explicitly stated so. For each brain, left and right ALs, OLs, and MBs were included as separate samples, resulting in 12 measurements per caste for these three regions and 6 measurements per caste for the CB. Significant differences between caste phenotypes in the relative volume of each brain region were tested for in R using a one-way ANOVA followed by Tukey’s post-hoc test. In all cases, *p*-values of <0.05 were interpreted as significant.

## References

[1] Kotrschal A., Rogell B., Bundsen A., Svensson B., Zajitschek S., Brännström I., Immler S., Maklakov A.A., Kolm N. (2013). Artificial selection on relative brain size in the guppy reveals costs and benefits of evolving a larger brain. Curr. Biol..

[2] Kotrschal A., Corral-Lopez A., Kolm N. (2019). Large brains, short life: selection on brain size impacts intrinsic lifespan. Biol. Lett..

[3] Herculano-Houzel S. (2011). Scaling of brain metabolism with a fixed energy budget per neuron: implications for neuronal activity, plasticity and evolution. PLoS One.

[4] Borst A. (2009). Drosophila's view on insect vision. Curr. Biol..

[5] Hansson B.S., Stensmyr M.C. (2011). Evolution of insect olfaction. Neuron.

[6] O'Donnell S., Clifford M.R., DeLeon S., Papa C., Zahedi N., Bulova S.J. (2013). Brain size and visual environment predict species differences in paper wasp sensory processing brain regions (Hymenoptera: Vespidae, Polistinae). Brain Behav. Evol..

[7] Sheehan Z.B.V., Kamhi J.F., Seid M.A., Narendra A. (2019). Differential investment in brain regions for a diurnal and nocturnal lifestyle in Australian Myrmecia ants. J. Comp. Neurol..

[8] Stöckl A., Heinze S., Charalabidis A., El Jundi B., Warrant E., Kelber A. (2016). Differential investment in visual and olfactory brain areas reflects behavioural choices in hawk moths. Sci. Rep..

[9] Heinze S., Reppert S.M. (2012). Anatomical basis of sun compass navigation I: The general layout of the monarch butterfly brain. J. Comp. Neurol..

[10] Keesey I.W., Grabe V., Gruber L., Koerte S., Obiero G.F., Bolton G., Khallaf M.A., Kunert G., Lavista-Llanos S., Valenzano D.R. (2019). Inverse resource allocation between vision and olfaction across the genus Drosophila. Nat. Commun..

[11] Rozanski A.N., Cini A., Lopreto T.E., Gandia K.M., Hauber M.E., Cervo R., Uy F.M.K. (2022). Differential investment in visual and olfactory brain regions is linked to the sensory needs of a wasp social parasite and its host. J. Comp. Neurol..

[12] Sulger E., McAloon N., Bulova S.J., Sapp J., O'Donnell S. (2014). Evidence for adaptive brain tissue reduction in obligate social parasites (Polyergus mexicanus) relative to their hosts (Formica fusca). Biol. J. Linn. Soc. Lond..

[13] Yan H., Simola D.F., Bonasio R., Liebig J., Berger S.L., Reinberg D. (2014). Eusocial insects as emerging models for behavioural epigenetics. Nat. Rev. Genet..

[14] Groh C., Ahrens D., Rössler W. (2006). Environment-and age-dependent plasticity of synaptic complexes in the mushroom bodies of honeybee queens. Brain Behav. Evol..

[15] Groh C., Rössler W. (2008). Caste-specific postembryonic development of primary and secondary olfactory centers in the female honeybee brain. Arthropod Struct. Dev..

[16] Roat T.C., da Cruz Landim C. (2008). Temporal and morphological differences in post-embryonic differentiation of the mushroom bodies in the brain of workers, queens, and drones of Apis mellifera (Hymenoptera, Apidae). Micron.

[17] Gordon D.G., Zelaya A., Arganda-Carreras I., Arganda S., Traniello J.F.A. (2019). Division of labor and brain evolution in insect societies: Neurobiology of extreme specialization in the turtle ant Cephalotes varians. PLoS One.

[18] Ehmer B., Gronenberg W. (2004). Mushroom body volumes and visual interneurons in ants: comparison between sexes and castes. J. Comp. Neurol..

[19] O’Donnell S., Bulova S., Barrett M. (2022). Brain plasticity indicates key cognitive demands in an animal society: caste comparisons in dampwood termites. Insectes Sociaux.

[20] Pahlke S., Jaumann S., Seid M.A., Smith A.R. (2019). Brain differences between social castes precede group formation in a primitively eusocial bee. Naturwissenschaften.

[21] O’Donnell S., Bulova S.J., DeLeon S., Barrett M., Fiocca K. (2017). Caste differences in the mushroom bodies of swarm-founding paper wasps: implications for brain plasticity and brain evolution (Vespidae, Epiponini). Behav. Ecol. Sociobiol..

[22] O’Donnell S., Bulova S., Barrett M., von Beeren C. (2018). Brain investment under colony-level selection: soldier specialization in Eciton army ants (Formicidae: Dorylinae). BMC Zool..

[23] Muscedere M.L., Traniello J.F.A. (2012). Division of labor in the hyperdiverse ant genus Pheidole is associated with distinct subcaste-and age-related patterns of worker brain organization. PLoS One.

[24] Fahrbach S.E., Moore D., Capaldi E.A., Farris S.M., Robinson G.E. (1998). Experience-expectant plasticity in the mushroom bodies of the honeybee. Learn. Mem..

[25] Maleszka J., Barron A.B., Helliwell P.G., Maleszka R. (2009). Effect of age, behaviour and social environment on honey bee brain plasticity. J. Comp. Physiol..

[26] Muratore I.B., Fandozzi E.M., Traniello J.F.A. (2022). Behavioral performance and division of labor influence brain mosaicism in the leafcutter ant Atta cephalotes. J. Comp. Physiol..

[27] Arganda S., Hoadley A.P., Razdan E.S., Muratore I.B., Traniello J.F.A. (2020). The neuroplasticity of division of labor: worker polymorphism, compound eye structure and brain organization in the leafcutter ant Atta cephalotes. J. Comp. Physiol..

[28] Bouchebti S., Arganda S. (2020). Insect lifestyle and evolution of brain morphology. Curr. Opin. Insect Sci..

[29] Lainé L.V., Wright D. (2003). The life cycle of Reticulitermes spp.(Isoptera: Rhinotermitidae): What do we know?. Bull. Entomol. Res..

[30] Tian L., Zhou X. (2014). The soldiers in societies: defense, regulation, and evolution. Int. J. Biol. Sci..

[31] Katoh H., Matsumoto T., Miura T. (2007). Alate differentiation and compound-eye development in the dry-wood termite Neotermes koshunensis (Isoptera, Kalotermitidae). Insectes Soc..

[32] Ishikawa Y., Aonuma H., Sasaki K., Miura T. (2016). Tyraminergic and octopaminergic modulation of defensive behavior in termite soldier. PLoS One.

[33] O’Donnell S., Bulova S., Barrett M. (2021). Experience-expectant brain plasticity corresponds to caste-specific abiotic challenges in dampwood termites (Zootermopsis angusticollis and Z. nevadensis). Naturwissenschaften.

[34] Ishibashi T., Waliullah A.S.M., Aramaki S., Kamiya M., Kahyo T., Nakamura K., Tasaki E., Takata M., Setou M., Matsuura K. (2023). Plastic brain structure changes associated with the division of labor and aging in termites. Dev. Growth Differ..

[35] Valadares L., da Silva I.B., Costa-Leonardo A.M., Sandoz J.-C. (2023). Differentiation of workers into soldiers is associated with a size reduction of higher-order brain centers in the neotropical termite Procornitermes araujoi. Sci. Rep..

[36] Thorne B., Traniello J., Adams E., Bulmer M. (1999). Reproductive dynamics and colony structure of subterranean termites of the genus*Reticulitermes*(Isoptera Rhinotermitidae): a review of the evidence from behavioral, ecological, and genetic studies. Ethol. Ecol. Evol..

[37] Fahrbach S.E. (2006). Structure of the mushroom bodies of the insect brain. Annu. Rev. Entomol..

[38] Crosland M.W., Su N.-Y. (2006). Work allocation among castes in a rhinotermitid termite (Isoptera) - Are nymphs a working caste?. Sociobiology.

[39] Chouvenc T., Ban P.M., Su N.-Y. (2022). Life and death of termite colonies, a decades-long age demography perspective. Front. Ecol. Evol..

[40] Seeley T.D. (1982). Adaptive significance of the age polyethism schedule in honeybee colonies. Behav. Ecol. Sociobiol..

[41] Farris S.M., Robinson G.E., Fahrbach S.E. (2001). Experience-and age-related outgrowth of intrinsic neurons in the mushroom bodies of the adult worker honeybee. J. Neurosci..

[42] Su N.-Y., Scheffrahn R.H., Cabrera B.J. (2001).

[43] Miura T., Matsumoto T. (1996). Ergatoid reproductives in Nasutitermes takasagoensis (Isoptera: Termitidae). Sociobiology.

[44] Thorne B.L., Noirot C. (1982). Ergatoid reproductives in Nasutitermes corniger (Motschulsky) (Isoptera: Termitidae). Int. J. Insect Morphol. Embryol..

[45] Noirot C., Thorne B.L. (1988). Ergatoid reproductives in Nasutitermes columbicus (Isoptera, Termitidae). J. Morphol..

[46] Watson J.A.L., Metcalf E.C., Sewell J.J. (1975). Preliminary studies on the control of neotenic formation in Mastotermes darwiniensis Froggatt (Isoptera). Insectes Soc..

[47] Myles T.G. (1999). Review of secondary reproduction in termites (Insecta: Isoptera) with comments on its role in termite ecology and social evolution. Sociobiology.

[48] Maekawa K., Mizuno S., Koshikawa S., Miura T. (2008). Compound eye development during caste differentiation in the termite Reticulitermes speratus (Isoptera: Rhinotermitidae). Zoolog. Sci..

[49] Paulk A.C., Dacks A.M., Phillips-Portillo J., Fellous J.-M., Gronenberg W. (2009). Visual processing in the central bee brain. J. Neurosci..

[50] Otsuna H., Ito K. (2006). Systematic analysis of the visual projection neurons of Drosophila melanogaster. I. Lobula-specific pathways. J. Comp. Neurol..

[51] Ito K., Shinomiya K., Ito M., Armstrong J.D., Boyan G., Hartenstein V., Harzsch S., Heisenberg M., Homberg U., Jenett A. (2014). A systematic nomenclature for the insect brain. Neuron.

[52] Honkanen A., Adden A., da Silva Freitas J., Heinze S. (2019). The insect central complex and the neural basis of navigational strategies. J. Exp. Biol..

[53] Kononenko N.L., Wolfenberg H., Pflüger H.J. (2009). Tyramine as an independent transmitter and a precursor of octopamine in the locust central nervous system: an immunocytochemical study. J. Comp. Neurol..

[54] Ishikawa Y., Aonuma H., Miura T. (2008). Soldier-specific modification of the mandibular motor neurons in termites. PLoS One.

[55] Roisin Y., Abe T., Bignell D., Higashi M. (2000). Termites: evolution, sociality, symbioses, ecology.

[56] Bourguignon T., Hayashi Y., Miura T. (2012). Skewed soldier sex ratio in termites: testing the size-threshold hypothesis. Insectes Soc..

[57] Matsuura K. (2006). Early Emergence of Males in the Termite <I>Reticulitermes speratus</I> (Isoptera: Rhinotermitidae): Protandry as a Side Effect of Sexual Size Dimorphism. Ann. Entomol. Soc. Am..

[58] Chouvenc T. (2019). The relative importance of queen and king initial weights in termite colony foundation success. Insectes Soc..

[59] Miura T., Matsumoto T. (1998). Open-air litter foraging in the nasute termite Longipeditermes longipes (Isoptera: Termitidae). J. Insect Behav..

[60] Miura T., Matsumoto T. (1998). Foraging organization of the open-air processional lichenfeeding termite Hospitalitermes (Isoptera, Termitidae) in Borneo. Insectes Sociaux.

[61] Haifig I., Jost C., Fourcassié V., Zana Y., Costa-Leonardo A.M. (2015). Dynamics of foraging trails in the Neotropical termite Velocitermes heteropterus (Isoptera: Termitidae). Behav. Processes.

[62] Raina A., Park Y.I., Florane C. (2003). Behavior and reproductive biology of the primary reproductives of the Formosan subterranean termite (Isoptera: Rhinotermitidae). Sociobiology.

[63] Ferreira M.T., Scheffrahn R.H. (2011). Light attraction and subsequent colonization behaviors of alates and dealates of the West Indian drywood termite (Isoptera: Kalotermitidae). Fla. Entomol..

[64] Bremer S., Hertel H., Wachmann E. (1993). Degeneration of the compound eye of the termite Neotermes jouteli (Isoptera) in darkness during the phase of reproduction. Zoomorphology.

[65] Crosland M., Lok C., Wong T., Shakarad M., Traniello J. (1997). Division of labour in a lower termite: The majority of tasks are performed by older workers. Anim. Behav..

[66] Crosland M.W.J., Traniello J.F.A. (1997). Behavioral plasticity in division of labor in the lower termite Reticulitermes fukienensis. Naturwissenschaften.

[67] Sun Q., Hampton J.D., Merchant A., Haynes K.F., Zhou X. (2020). Cooperative policing behaviour regulates reproductive division of labour in a termite. Proc. Biol. Sci..

[68] Zhou C., Pan Y., Robinett C.C., Meissner G.W., Baker B.S. (2014). Central brain neurons expressing doublesex regulate female receptivity in Drosophila. Neuron.

